# Neonatal Surveillance Gaps in Maternal Graves' Disease: Findings From a Queensland Maternity Hospital

**DOI:** 10.1111/ajo.70140

**Published:** 2026-04-30

**Authors:** Umesha Pathmanathan, Annabel Wingate, Stephanie Teasdale

**Affiliations:** ^1^ Obstetric Medicine Department Mater Misericordiae Ltd South Brisbane Queensland Australia; ^2^ Griffith University School of Medicine and Dentistry Southport Queensland Australia

## Abstract

**Background:**

Graves' disease presents unique challenges in pregnancy due to the risk of neonatal morbidity and mortality from maternal TSH Receptor antibodies (TRAb). A maternity hospital in Queensland has implemented a policy to identify at‐risk neonates and facilitate follow‐up, including cord blood TRAb, thyroid function tests (TFTs), and clinic appointments.

**Aims:**

To evaluate current neonatal follow‐up practices for mothers with Graves' disease, assess missed opportunities for biochemical testing and propose recommendations to improve clinical care.

**Materials and Methods:**

This retrospective audit covered deliveries between 1st January 2018 to 31st December 2022. Baseline characteristics and follow‐up were summarised by frequency and percentage. Maternal records were reviewed for demographic data, ultrasound follow up, TRAb titres, TFTs, and treatment details. Corresponding neonatal charts were then reviewed for cord blood TRAb levels, TFTs on days 3–5 and days 10–14, and phone clinic appointments. Neonatal testing was indicated if the mother was positive for thyroid stimulating immunoglobulin (TSI) or if TRAb titres were > 3 times the upper limit of normal.

**Results:**

Data from 70 mothers with active or previously treated Graves' disease were analysed; 18 pregnancies met criteria for neonatal testing. Compliance with recommended testing was suboptimal, with variable adherence to TFT testing and only 10% undergoing cord blood TRAb measurement.

**Conclusion:**

This audit highlights the importance of standardised protocols and ongoing education to optimise neonatal follow‐up and improve outcomes for neonates born to mothers with Graves' disease. Recommendations for education, improved documentation and policy updates have been provided with the aim for further audits to ensure ongoing quality improvement.

## 1 Introduction

Graves' disease is the most common cause of autoimmune hyperthyroidism and affects 0.2% of pregnancies [1]. Neonates born to mothers with Graves' disease are at risk of significant morbidity and mortality in the context of transplacental transfer of maternal thyroid receptor antibodies (TRAb) from the second trimester of pregnancy [2]. This can induce foetal or neonatal thyrotoxicosis, resulting in tachycardia and other cardiac arrhythmias leading to cardiac failure, foetal goitre formation and growth restriction, preterm delivery, microcephaly, premature bone ossification, psychomotor disabilities, and craniosynostosis [3, 4, 5, 6]. Capturing thyroid disturbance early is imperative for treatment and reducing the risk of intellectual impairment [7]. Therefore, several guidelines have been established based on the literature to identify neonates who are at high risk of complications.

Van de Kaay et al. [2] recommends measuring TRAb levels between 20 and 24 weeks gestation. The American Thyroid Association (ATA) and Endocrine Society have recognised that the risk of thyrotoxicosis is highest when the TRAb level is above two to three times the upper limit of normal (ULN) and requires close foetal and neonatal monitoring [7, 8]. High titres are present not only in active Graves' disease, but also in euthyroid women who have been previously treated with radioactive iodine or thyroidectomy [9]. However, Tzoraki et al. [10] highlighted the lack of a standardised protocol for neonatal screening within the first week of life, contributing to variable outcomes and the potential for missed diagnoses of thyroid dysfunction.

A cohort study by Priyanka et al. [11] demonstrated a strong correlation between maternal TRAb levels in the third trimester and corresponding neonatal levels. Positive neonatal TRAb levels were associated with transient thyroid dysfunction, including thyrotoxicosis in 31.8% of cases. Similar findings were reported by Besancon et al. [12] in a prospective observational study of 68 neonates: none of the neonates born to TRAb‐negative mothers developed hyperthyroidism, while 72.7% of those with positive cord blood TRAb results were at risk, and 29.2% developed neonatal hyperthyroidism. These findings support routine maternal thyroid function tests (TFTs) and TRAb testing in women with a history of Graves' disease and serial foetal ultrasounds, cord blood testing for TRAb at delivery, and neonatal testing of TFTs at days 3 to 5 of life to facilitate timely diagnosis and intervention [1, 12, 13, 14, 15, 16].

Retrospective audits further highlight the variability in current practice and the risk of underdiagnosis. Luz et al. [17] found that cord blood TRAb levels were valuable predictors of neonatal thyroid dysfunction and raised concerns of inconsistent postnatal monitoring potentially resulting in missed or late diagnoses. Ben‐Zeev et al. [18] similarly noted a lack of uniform testing in their cohort, stressing the importance of both cord blood TRAb and neonatal TFTs in identifying at‐risk infants.

In response to the literature and previous experience, an expert panel comprising obstetricians, obstetric physicians, endocrinologists and neonatologists at a tertiary maternity hospital developed a consensus guideline in 2017 addressing maternal thyroid dysfunction, including specific recommendations for neonatal follow‐up in maternal Graves' disease. The protocol outlines risk stratification based on maternal TRAb status, indications for cord blood TRAb and serial TFT testing, scheduled follow‐up, and criteria for urgent escalation if neonatal thyrotoxicosis is suspected.

This audit was completed in response to an internal review, which raised concerns of inconsistent screening practices despite these guidelines. While many clinical guidelines outline the recommended management of such pregnancies, few studies have systematically assessed real‐world implementation or examined the barriers to consistent application. Furthermore, there is limited published evidence regarding the uptake and adherence of these protocols within the Australian maternity care settings.

The aims and objectives of this audit were to:

1. Assess the current clinical practice regarding maternal biochemical testing and neonatal follow‐up, and compare this against existing local and international guidelines.
2. Identify gaps in practice and provide evidence‐based recommendations to guide future improvements in clinical care.

## 2 Materials and Methods

A retrospective audit was conducted. Individuals who received antenatal care and delivered at the hospital between 1st January 2018 to December 31st 2022 were identified using ICD‐10‐AM codes and electronic health databases including VERDI and Matrix. Search terms included hyperthyroidism, Graves' Disease, hypothyroidism, thyroid disease and thyroid nodule.

Maternal charts were reviewed to confirm a diagnosis of Graves' disease for inclusion in the final dataset. Demographic data such as ethnicity and gestational age at delivery were recorded. Maternal thyroid function tests (TSH, fT3 and fT4) were extracted. Active Graves' disease was defined as mothers with evidence of subclinical (TSH suppression with normal free T4) or overt hyperthyroidism (TSH suppression with high free T3 or free T4), with a known history of Graves' disease or treatment with antithyroid medications during pregnancy. Treatment details and ultrasound monitoring were also noted.

Thyroid antibody testing was reviewed, including TRAb or thyroid stimulating immunoglobulin (TSI) performed in the second trimester. A positive antibody result was defined as a TRAb titre > 3 times the lab‐specific upper limit of normal or a TSI above the upper limit of normal. If both antibodies were tested and discordant results were noted, it was defined as positive. An equivocal result was based on the laboratory‐specific reference ranges in which the result was unable to be interpreted as either positive or negative.

Neonatal charts were also reviewed to evaluate cord blood TRAb levels and thyroid function tests on day 3–5 and days 10–14 of life, as per the policy. Additionally, a review was conducted to check if a phone clinic appointment had been completed to follow up on any pending results in the neonatal charts.

Data was analysed utilising descriptive statistics. Baseline characteristics and follow up were summarised by frequency and percentage.

## 3 Results

70 women were identified to have active or previously treated Graves' disease. 35 women (50%) had active Graves' disease during the pregnancy, with 27 women on antithyroid medications during pregnancy. Overall, 42 (60%) of mothers were euthyroid, 12 (17%) were hyperthyroid, and 15 (21%) had subclinical hyperthyroidism. Table 1 demographics are demonstrated below.

**TABLE 1:** Patient Demographics.

	Number	Percentage %
Ethnicity		
Aboriginal/Torres Strait Islander	2	2.8
South Asian	7	10
East Asian	25	35.7
Caucasian	26	37.1
African	3	4.2
Other	7	10
Graves' Disease		
Active	35	50
Previous Active	35	50
Treatment		
None	33	47.1
Anti‐thyroid Drugs	27	38.5
Thyroidectomy	7	10
Radioactive Ablation	3	4.3
Gestational Age at Delivery		
< 30 weeks	0	0
30–34 weeks	2	2.8
34–37 weeks	8	11.4
> 37 weeks	60	85.7
Serial Ultrasound Assessments		
None	30	42.9
1–2 scans	19	27.1
4‐weekly	21	38.6
Goitre Present	6	8.6

63 mothers (90%) who had a diagnosis of Graves' disease had an available antibody result. 70% had TRAb testing, 22% had TSI testing, and 7% both. Of the 5 mothers who had both tested, only 1 had a discordant result with TSI positivity 0.87 IU/L (< 0.55 IU/L) and TRAb negativity. Table 2 highlights the antibody results. All the untested population belonged in the previously treated Graves' disease category; 100% of this population were euthyroid at the initial assessment. 1 (16%) of the untested population had been previously treated with thyroidectomy and was subsequently found to be TSI positive in the postpartum period.

**TABLE 2:** Maternal Thyroid Antibody Results.

	Number (%)	TRAb or TSI tested (% of number)	TRAb (% of tested)	TSI (% of tested)	TRAB > 3× ULN	TSI positive	Positive (overall)
Overall	70	63 (90%)	44 (70%)	14 (22%)	12 (17%)	8 (11%)	20 (29%)
Active Graves' Disease	29	35 (100%)	19 (54%)	12 (34%)	12 (100%)	6 (75%)	18 (90%)
No treatment	7	7 (100%)	3 (16%)	3 (25%)	3 (25%)	0 (0%)	3 (17%)
Anti‐thyroid drugs	27	27 (100%)	15 (79%)	9 (75%)	8 (67%)	6 (100%)	14 (78%)
Thyroidectomy	1	1 (100%)	1 (5%)	0 (0%)	1 (8%)	0 (0%)	(1 6%)
Previously treated Graves' Disease	35 (50%)	28 (80%)	25 (89%)	2 (7%)	0 (0%)	2 (25%)	2 (10%)
No current treatment	26	20 (77%)	17 (68%)	2 (100%)	0 (0%)	1 (50%)	1 (50%)
Thyroidectomy	6	5 (83%)	5 (20%)	0 (0%)	0 (0%)	0 (0%)	1 (50%)
Radioactive Ablation	3	3 (100%)	3 (12%)	0 (0%)	0 (0%)	1 (50%)	0 (100%)

Abbreviations: TRAb, maternal thyroid receptor antibodies; TSI, thyroid simulating immunoglobulin; ULN, upper limit of normal.

The protocol delineates cord blood for patients with TRAb positivity with D3‐5 TFTs testing and then D10 testing if indicated. TSI positive patients also followed similar testing pattern. 18 pregnancies were included for analysis, with 2 sets of twins totalling to 20 neonates. In those neonates, 2 (10%) had testing of cord blood TRAb, 55% had D3‐5 TFTs and 70% had D10‐14 TFTs. 70.5% also had a phone clinic review to chase results. Overall compliance was 2 (10%) of neonates with the protocol. The results are seen below in (Table 3).

**TABLE 3:** Neonatal Thyroid Antibody Testing.

	TRAb > 3× ULN	TSI positive	Both
Total	12	8	20
Serial USS	9 (75%)	3 (38%)	12 (60%)
Cord Blood Taken	6 (50%)	5 (63%)	11 (55%)
Cord Blood TRAb	0 (0%)	2 (25%)	2 (10%)
Peripheral TRAb	1 (10%)	0 (0%)	1 (5%)
NST Done	10 (83%)	6 (75%)	16 (80%)
Early TFTs	3 (25%)	2 (25%)	5 (25%)
TFTs D3‐D5	7 (58%)	6 (75%)	13 (65%)
TFTs D10‐D14	8 (67%)	8 (100%)	16 (80%)
Phone Clinic Reviews			
Total Required	11 (92%)	7 (88%)	18 (90%)
Phone Clinic Completed	7 (58%)	6 (86%)	13 (65%)
Total Compliance	0 (0%)	2 (10%)	2 (10%)

Abbreviations: NST, neonatal screening test; TFTs, thyroid function tests; TRAb, thyroid receptor antibodies; ULN, upper limit of normal; USS, Ultrasound.

**FIGURE 1:**
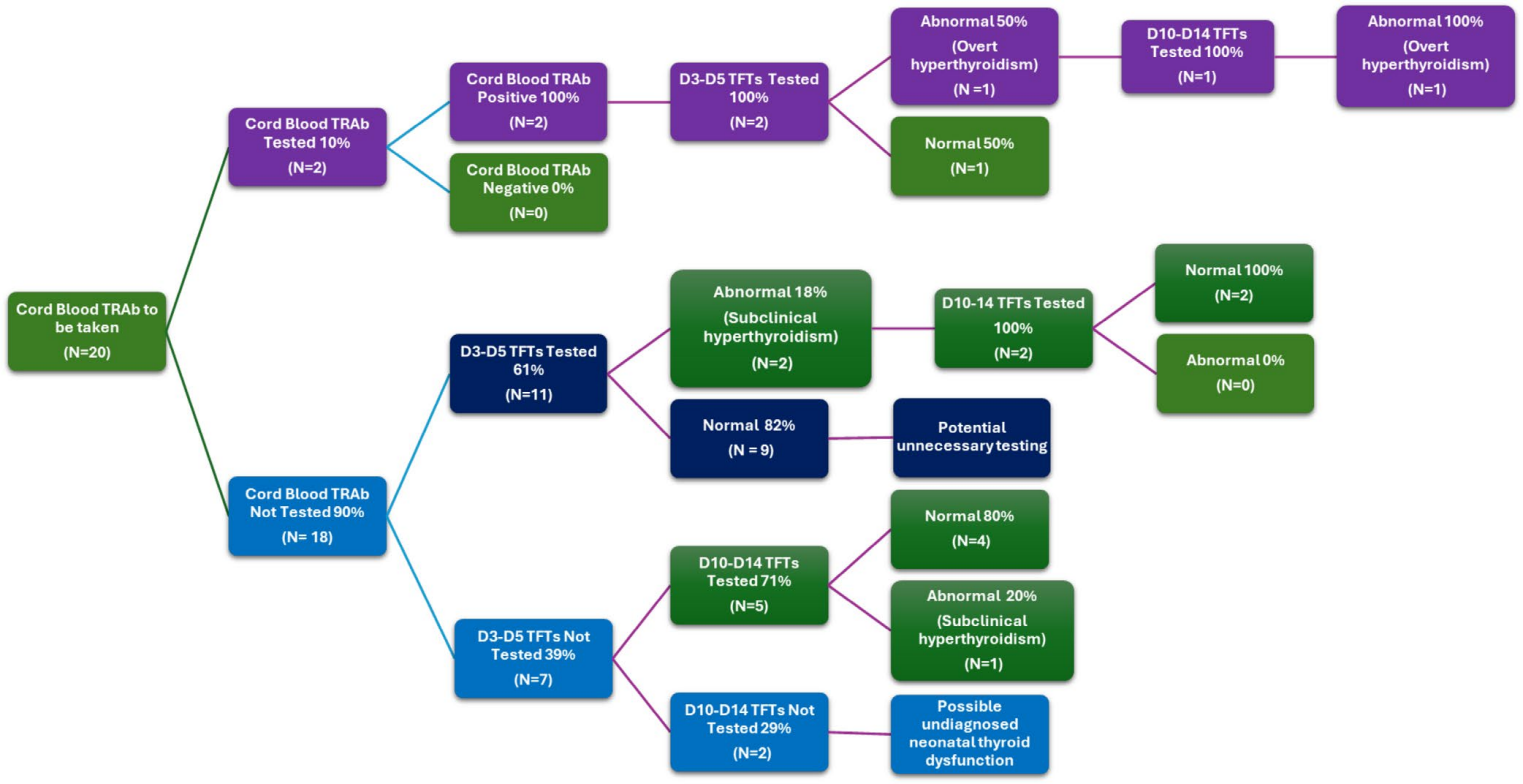
Relationship between Cord Blood Sampling and Thyroid Function Tests at Days 3–5 and Days 10–14. TRAb: Thyroid receptor antibodies, TFTs: Thyroid function tests, D3‐D5: Days 3–5, D10‐14: Days 10–14.

Figure 1 demarcates the abnormalities in neonatal thyroid function in comparison to cord blood sampling in those mothers who met the criteria. Of the 2 neonates appropriately tested, 1 was found to be hyperthyroid requiring follow up and treatment in a tertiary paediatric facility.

Of the not tested group, 61% went on to have D3‐D5 TFT testing, of which 2 (18%) were found to have subclinical hyperthyroidism. The TFTs were tested and had normalised at subsequent D10‐14 testing. 1 neonate was found to have abnormal TFTs after day 10 of birth, which was subclinical hypothyroidism. The mother was euthyroid with previously treated Graves' disease not on antithyroid medication; however, she did not have antibody testing during the pregnancy.

## 4 Discussion

This audit aimed to evaluate the compliance of clinical practice with the institutional policy for managing neonates born to mothers with Graves' disease at a Queensland maternity hospital. Between 2018 and 2023, there were a total of 31 318 births, and the sample size for this audit was in line with the expected prevalence of Graves' disease in pregnancy of approximately 0.2% [1, 19].

The findings demonstrated suboptimal compliance, particularly with cord blood TRAb testing which was only performed in 10% of eligible cases, despite its well‐established role in predicting the risk of neonatal hyperthyroidism. Multiple studies have shown that elevated TRAb levels in cord blood are associated with an increased risk of neonatal hyperthyroidism during the first 2 weeks of life, whereas negative TRAb levels are generally linked to minimal to no risk [2, 12, 14, 20, 21]. Due to their high sensitivity, negative cord blood TRAb results can safely justify discharge without further thyroid function monitoring, and this approach has been incorporated into current clinical protocols [2, 14, 20, 22]. In our cohort, TFT testing may have been unnecessary in the presence of a negative TRAb result, indicating potential resource overuse and patient burden.

The compliance rate to clinical guidelines is variable in the previous literature. Cano et al. [13] prospectively followed neonates born to mothers with active or previous Graves' disease, using cord blood TRAb testing when maternal titres were positive in the second trimester. Of the 33 neonates included, two were identified as at risk, though neither developed thyroid dysfunction. Antenatal testing compliance was higher (84.4%) than at birth (75.8%). Another prospective study of 20 women with Graves' disease showed poor adherence to the local screening protocols, with only one infant undergoing cord blood testing at birth. However, follow‐up testing rates improved to 68.4% at 48 h and 89.5% at day seven [23]. In contrast, a larger study of 72 women reported 100% compliance with cord blood testing, identifying six TRAb‐positive neonates who all developed hyperthyroidism [24].

Cord blood TRAb testing is often limited by test availability and prolonged turnaround times, which may delay results for several days. Furthermore, reduced compliance may stem from the current responsibility for sample collection falling on the obstetrics team, prior to neonatal speciality involvement. Additional contributing factors include inadequate staff education, inconsistent documentation in the maternity care plan and electronic systems such as Matrix, and the lack of clear role delineation. These issues reflect common root causes for suboptimal implementation of clinical practice guidelines, as highlighted in a systematic review in the primary care setting by Wang et al. [25].

To enhance adherence, a targeted quality improvement (QI) approach should focus on enhancing the clarity and access to the guideline documentation, leveraging local opinion leaders to drive clinical engagement, and addressing environmental and workflow barriers that impede adherence [26]. Strategies may include: training sessions for the relevant clinical teams, integrating electronic reminders into the patient's electronic record, and implementing real‐time alerts for pregnancies at high risk of neonatal thyrotoxicosis [27]. Although existing hospital policy advises documenting clinical information into Matrix and the patient's maternity care plan, compliance with this requirement should be audited. Environmental and workflow obstacles must also be considered. Assigning responsibility for documentation to the obstetric medicine team—who oversees Graves' disease management—may enhance accountability and follow‐through. Additionally, the use of local opinion leaders to promote best practices from a senior clinician level and incorporating the protocol into routine handover discussions can also further support sustainable practice change.

The confusion surrounding TRAb versus TSI testing further complicates adherence. The hospital policy discourages TSI testing in pregnancy due to the absence of validated pregnancy‐specific reference ranges. However, many pathology providers have transitioned to using TSI assays exclusively, citing increased sensitivity. TSI is indeed a more specific marker for stimulating antibodies compared to TRAb, and has been associated with a higher risk of neonatal hyperthyroidism [28]. A retrospective study by Cui & Rijhsinghani [29] found that a TSI > 2.5 times the upper limit of normal correlated with increased neonatal risk, while another study found all hyperthyroid neonates had TSI > 5 IU/L [30] In our cohort, the most significantly elevated cord TRAb levels were observed in neonates whose mothers had extremely high TSI titres (> 25× ULN). Given the poor compliance with cord blood testing and small sample size, these findings require further validation. Currently, there is no clear policy guidance for interpretation or follow up of TSI results in pregnancy, and this diagnostic ambiguity likely contributes to under‐testing. Future revisions of the protocol would benefit from including criteria for TSI follow‐up and interpretation.

Another challenge was the lack of defined timing for TRAb testing in pregnancy. In our cohort, the TRAb result in the second trimester was used. Existing guidelines recommend TRAb testing in hyperthyroidism or with a known or previous history of Graves' disease in early pregnancy, and then again between 18 to 22 weeks and 28 to 34 weeks if elevated or the patient is being treated with antithyroid medication [7]. 90% were appropriately tested in this study. High titres in the third trimester are most strongly associated with neonatal thyrotoxicosis risk [7, 20, 28]. Therefore, inconsistent TRAb testing could lead to under‐recognition of neonates at risk. A future QI initiative could involve revising the current policy to clearly define the recommended timing for TRAb testing during pregnancy. Additionally, auditing TRAb testing by gestational age could help assess adherence to guidelines and identify opportunities to optimise the timing for maximal clinical relevance.

While this audit was conducted in a single tertiary centre, the findings are applicable to other institutions managing high‐risk pregnancies with autoimmune thyroid disease. The barriers identified including the lack of clarity around testing responsibility, limited understanding of protocol specifics, and inconsistent documentation are common across many clinical settings. These system‐level issues necessitate not only clinical guidelines but robust implementation strategies grounded in QI methodology. The main limitation of this audit was the small sample size; future reviews would benefit from a multi‐centre approach to enhance generalisability and strengthen the validity of the findings.

Based on these findings, the following recommendations have been proposed:

1. Clarify and standardise the policy

- Update the policy to define the timing of TRAb testing during pregnancy.
- Include guidance on TSI interpretation and define follow‐up actions for elevated TSI levels.

2 Improve documentation processes

- Standardise documentation templates in the maternity care plan and Matrix to include a designated section for TRAb/TSI screening and neonatal testing plans.
- Assign responsibility for documenting to the obstetric medicine team.
- Audit documentation to ensure completeness.

3 Education and engagement

- Deliver targeted education sessions to obstetric, obstetric medicine, and neonatal teams on updated protocol, testing responsibilities, and clinical rationale for TRAb/TSI monitoring.
- Engage local opinion leaders (senior obstetricians, obstetric physicians, and neonatologists) to reinforce adherence.

4 Audit and Feedback

- Conduct regular audits of TRAb/TSI testing, neonatal testing compliance, and documentation quality.
- Introduce qualitative surveys to understand provider barriers.
- Provide real‐time feedback to teams to support accountability.

5 Implement iterative PDSA cycles

- Commence the next PDSA cycle to implement the revised policy and educational interventions.

This audit identified significant gaps between clinical practice and institutional protocol in the management of neonates at risk for thyroid dysfunction due to maternal Graves' disease. While policy exists, implementation is lacking. A repeat audit and engagement with frontline clinicians will be crucial in ensuring sustained improvement and patient safety.

## Ethics Statement

Ethics approval given by the Mater Human Research Ethics Committee.

## Conflicts of Interest

The authors declare no conflicts of interest.

## Data Availability

The data that support the findings of this study are available on request from the corresponding author. The data are not publicly available due to privacy or ethical restrictions.
